# Clinical Results in Patients with Combined Penetrating Keratoplasty and Vitreoretinal Surgery Using Landers Wide-field Temporary Keratoprosthesis

**DOI:** 10.4274/tjo.galenos.2019.87059

**Published:** 2019-10-24

**Authors:** Hüseyin Mayalı, Özcan Kayıkçıoğlu, Muhammed Altınışık, Faruk Bıçak, Emin Kurt

**Affiliations:** 1 Manisa Celal Bayar University Faculty of Medicine, Department of Ophthalmology, Manisa, Turkey

**Keywords:** Temporary keratoprosthesis, pars plana vitrectomy, penetrating keratoplasty

## Abstract

**Objectives:**

To evaluate the clinical results of combined pars plana vitrectomy (PPV) with Landers wide-field temporary keratoprosthesis and penetrating keratoplasty (PK).

**Materials and Methods:**

From January 2016, traumatic eyes with coexisting corneal and vitreoretinal diseases that underwent combined keratoprosthesis/PPV/PK surgery were retrospectively evaluated. Demographic characteristics, visual acuity (VA), intraocular pressure (IOP) and clinical findings of the cornea, lens, and retina were recorded during the follow-up. Cases with clear corneal graft, attached retina, normotonic IOP, and improved or stable VA were considered successful.

**Results:**

Eight eyes were enrolled in the study. The mean follow-up time was 21.1±8.20 months. Surgery was performed a mean of 23 (10-40) days after trauma. Preoperative VA ranged from no light perception to counting fingers from 50 cm. Postoperatively, corneal graft was clear in 5 patients (62.5%) and retina was attached in 6 eyes (75%). Chronic hypotonia developed in 3 patients (37.5%). VA was unchanged in 3 patients and improved in 5 patients. A total of 5 cases (50%) were considered successful. Shorter interval between trauma and surgery was associated with higher likelihood of success (p=0.043). No significant difference was observed between the groups in terms of type or location of trauma (p=1; p=0.143).

**Conclusion:**

Although the functional results are not very satisfactory, the combined procedure provides a final opportunity for preserving remaining vision and anatomic reconstruction in eyes that will otherwise result in phthisis due to severe anterior and posterior segment pathologies.

## Introduction

Visualization of the posterior segment during pars plana vitrectomy (PPV) may be impeded by diffuse corneal edema secondary to ocular traumas, distortions due to suturation of large and irregular corneal lacerations, or corneal scars. For cases like these which were previously considered inoperable, we now have alternatives such as open sky vitrectomy, endoscopic PPV, or PPV surgeries using temporary keratoprosthesis.

**Figure 1:**
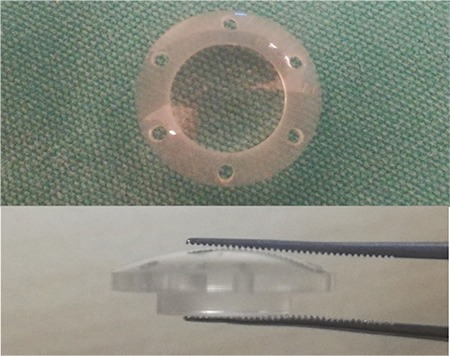
Landers wide-angle keratoprosthesis

Temporary keratoprostheses are auxiliary instruments that are temporarily sutured to the trepanized corneal bed to provide a clear view during PPV in eyes with an opaque cornea. The first keratoprostheses described by Landers in 1981 were biconcave instruments made of polymethylmethacrylate (PMMA) with a cylindrical optical body 5 mm in length.^1^ However, these keratoprostheses leaked, caused distortion, and had a narrow viewing field that made it difficult to see the anterior and peripheral retina, leading to the development of new interfaces. In 1993, the Landers wide-angle keratoprostheses with convex anterior surface and 1-mm cylindrical body were produced (Figure 1).^2^ Another alternative, Eckardt keratoprostheses, are made of silicone but were not superior to the Landers keratoprosthesis because they lacked durability over multiple uses.^3^

In the present study, we report our anatomical and functional outcomes of combined penetrating keratoplasty (PK) and PPV using the latest generation Landers keratoprosthesis.

## Materials and Methods

The study included traumatic patients who underwent the triple procedure of combined PK and PPV with Landers wide-angle keratoprosthesis (7.2 mm version, Ocular Instruments, Bellevue, USA) in the Manisa Celal Bayar University Department of Ophthalmology, a tertiary referral ophthalmology center, since January 2016.

This retrospective cross-sectional study was approved by the university ethics committee and conducted in accordance with the principles of the Declaration of Helsinki. Informed consent was obtained from the patients before surgery.

The patients’ demographic information (age, sex), follow-up time, findings in preoperative/postoperative full ophthalmological examinations, and postoperative complications (graft rejection, infection, glaucoma, phthisis, retinal detachment [RD]) were recorded.

Visual acuity (VA) could not be measured using Snellen chart, and were instead recorded as hand motion (HM), counting fingers (CF), light perception (LP), or no LP (NLP). Intraocular pressure (IOP) was measured using applanation tonometry. Patients with IOP between 8 and 21 mmHg were considered normotonic.

Eyes included in the study had corneal pathologies secondary to open globe injuries, accompanied by vitreoretinal pathologies. The patients underwent primary corneoscleral suturation as an emergent intervention. The combined triple procedure was performed as a secondary surgical procedure in eyes found to be normotonic in follow-up following primary suturation.

Primary endpoints of the study were defined as corneal graft transparency, retinal attachment, IOP (normotonic/hypotonic), and VA (increased, maintained or decreased).

Patients with VA of HM or better at last follow-up visit were considered to have functional vision.

Eyes with transparent graft, attached retina, normotonic IOP, and maintained or increased VA were classified as successful.

### Ocular Trauma Classification

Based on the Birmingham Eye Trauma Terminology^4^, trauma cases were categorized as rupture, perforation, and penetration. Based on the Ocular Trauma Classification (OTC), wound sites were classified as Zone 1 if limited to the cornea, Zone 2 if extending into the sclera within 5 mm posterior of the limbus, and Zone 3 if posterior to Zone 2.^5^

### Surgical Procedure

All surgeries were performed under general anesthesia by surgeons experienced in anterior segment surgery (H.M.) and vitreoretinal surgery (Ö.K.).

**Figure 2:**
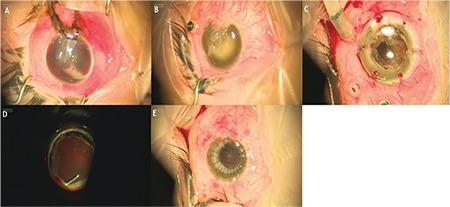
Stages of combined keratoprosthesis, pars plana vitrectomy, and penetrating keratoplasty surgery in a traumatic eye

A 23-gauge (G) sclerotomy was made in the lower temporal quadrant for infusion cannula placement. After connecting the infusion line (Ocrosol Balanced Salt Solution, Polifarma, Turkey), ocular tone was achieved (Figure 2A, B).

The recipient cornea was trepanned (Hessburg Barron, Jedmed Ltd, St. Louis, USA) and full-thickness excision was performed using microcorneal scissors. A 7.2-mm Landers wide-angle prosthesis was sutured to the corneal bed using 6-0 vicryl (Johnson & Johnson, USA) (Figure 2C). Anterior segment procedures such as cataract extraction, secondary intraocular lens (IOL) implantation, scleral fixation IOL implantation, and synechiolysis were performed when necessary.

Vitrectomy trocars, endoillumination probe, and chandelier light probe were introduced through pars plana sclerotomies and standard 4-port 23-G PPV surgery was performed (Constellation, Alcon, Fort Worth, TX, USA). An Elbos wide-angle imaging system (Möller-Wedel, Wedel, Germany) was used during surgery (Figure 2D). Core vitrectomy, vitreous base cleaning, and fibrovascular membrane cleaning were performed in all cases. Perfluorocarbon fluid (Teknomek, Istanbul, Turkey), endolaser photocoagulation (Oculight SC, IRIDEX, California), and relaxing retinectomy procedures were also employed when indicated. Silicone oil (Mersilicon 1000, Meran, Istanbul) was used as intraocular tamponade when indicated.

In the third stage of the surgery, the keratoprosthesis was removed and a corneal graft that was 0.5 mm larger than the recipient corneal bed and stored in McCarey–Kaufman medium was sutured to the recipient corneal bed using 16 individual 10-0 nylon sutures (Visionary Medical Supplies Inc. Madison, USA) (Figure 2E).

### Postoperative Care

Postoperative topical moxifloxacin (Vigamox, Alcon, Novartis Company, USA) and prednisolone acetate (Pred forte, Allergan, USA) were applied 8 times daily; cyclopentolate (Sikloplejin 1%, Abdi Ibrahim, Turkey) was applied 3 times daily. All patients continued to receive low-dose steroid (3 times daily) for at least a year.

Corneal graft transparency, VA, IOP, retinal attachment, and complications were evaluated in follow-up examinations.

### Statistical Analysis

Statistical analysis was performed using SPSS 24.0 for Windows. Data were recorded as mean ± standard deviation. Distribution pattern was determined based on Shapiro-Wilk test. Categorical variables were compared using Fisher’s exact test, numerical variables were compared using tests appropriate for their distribution patterns (Student’s t test for normal, Mann-Whitney U test for nonnormal distributions). A p value <0.05 was considered significant.

## Results

Table 1 summarizes the clinical and demographic characteristics of the patients. A total of 8 patients with open globe trauma were included in the study. Seven of the patients were male and 1 was female. The mean age of the cases was 47.50±15.91 years (16–64 years). The mean follow-up time was 21.13±8.2 months (3-28 months). Mean preoperative IOP was 12.88±4.05 mmHg (8-18 mmHg). In our study, the mean time from trauma to combined surgery was 23 days (10-40 days).

According to OTC, 6 of the cases were rupture and 2 were penetration. Wound sites were in Zone 2 in 3 cases and Zone 3 in 5 cases. The trauma patients who underwent combined surgery were those who underwent emergency primary corneoscleral suturation and were not hypotonic during follow-up.

In all cases, the intraocular structures could be visualized without distortion during surgery. The peripheral retina could be seen upon indentation. The keratoprosthesis did not leak during indentation. There were no intraoperative complications.

### Preoperative Findings

Two eyes exhibited diffuse corneal edema secondary to trauma. One eye had leukoma and another had hematic cornea. In the other 4 eyes, corneal anatomy was severely disrupted due to irregular suturation secondary to trauma (Table 1).

Evaluation of preoperative and intraoperative posterior segment pathologies revealed vitreous hemorrhage in all cases. Four eyes also had RD. Nucleus drop was seen in 3 eyes (Table 1).

VA before combined surgery ranged from NLP to CF from 50 cm. Vision level was LP in 5 eyes and HM, CF 50cm, and NLP in the other 3 eyes.

Silicone oil was used as a tamponade during surgery.

### Postoperative Findings

None of the corneal grafts showed postoperative wound leaks. At last follow-up visit, corneal graft failure was observed in 3 eyes (37.5%), while the other 5 eyes (62.5%) had transparent corneas. One eye underwent rekeratoplasty at 8 months due to infectious corneal ulcer, and the cornea was transparent at last follow-up (Patient 3). Early graft rejection (month 3) occurred in a young patient (Patient 6).

Retinal attachment was observed in 6 eyes (75%) during follow-up. Silicone was present in one of the eyes with attached retinas while it was removed in the other. Two eyes (25%) showed RD under the silicone.

The mean postoperative IOP of the eyes was 10±4.27 mmHg. In terms of complications, 3 eyes (37.5%) had chronic hypotony and 2 of those eyes resulted in phthisis. Proliferative vitreoretinopathy (PVR), macular atrophy, and graft rejection occurred in 1 eye each. One eye developed infectious corneal ulcer but had transparent graft in follow-up after the second PK.

VA at final visit was unchanged in 3 patients and improved in 5 patients. The greatest increase in VA was from LP to CF 20 cm in Patient 7. Functional vision (HM or better) was achieved in 6 cases (75%).

In total, 4 cases (50%) were considered complete success and 4 cases (50%) were considered failed. The demographic and clinical characteristics of the successful and failed cases are summarized in Table 2. In our study, successful trauma cases had significantly shorter mean time to surgery than failed cases (p=0.043). Success was not associated with type or location of trauma (p=1, p=0.143).

None of the cases had indications for enucleation or sympathetic ophthalmia.

## Discussion

In this series of patients with corneal opacification secondary to trauma and coexisting vitreoretinal pathologies, combined PPV and PK surgery performed with Landers wide-angle keratoprosthesis resulted in retinal attachment in 6 cases (75%), normotony in 5 cases (62.5%), and graft transparency in 5 cases (62.5%). VA did not decrease in any of the cases, increased in 5 cases (62.5%), and was unchanged in 3 cases. In total, 4 cases (50%) were considered completely successful.

Various alternatives have been used in attempts to perform posterior segment surgeries in eyes with corneal opacities. The main alternatives are performing PK followed by PPV in a separate session, performing simultaneous open sky vitrectomy with corneal excision, or performing PPV with endoscopic methods or temporary keratoprostheses.^6^

When PK and PPV surgeries are performed in separate sessions, PPV may be delayed due to risks such as persistent corneal edema or graft rejection. Moreover, it has been reported that fluid movements in the anterior chamber and ocular manipulations during PPV also risk damaging the corneal graft.^7^

In the open sky method, ensuring rotational eye movement and eye positioning is difficult, and there is risk of extracortical hemorrhage.^8^

Temporary keratoprostheses are auxiliary surgical instruments that enable posterior segment visualization in eyes with corneal opacities. These devices have been shown in the literature to allow all maneuvers without leaking any fluids during surgery.^8,9,10^ This technique is also recommended for the subacute management of massive ocular traumas.^11^ It has been found superior to other methods because it allows closed-system surgery, wide-angle stereoptic vision, and bimanual surgery.

Consistent with the literature, there were no complications in terms of peripheral vision, scleral indentation, or leaks during the surgeries utilizing the wide-angle Landers keratoprosthesis in this study.

In PK surgery, it has been reported that preparing a corneal graft 0.5 mm larger than the recipient bed prevents postoperative angle-closure glaucoma.^12^ In our case series, we used grafts of similar dimensions and encountered no problems.

Different results have been reported in the literature regarding anatomical reconstruction and visual gains after combined triple surgery with keratoprosthesis. The reported corneal graft survival rates are 25-79% and retinal attachment rates are 48-100%, while the proportion of postoperative normotonic eyes is 20-75%. In terms of VA, the proportion of eyes in the literature that achieve a functional level of vision is 25-75%.^7,8,13,14,15,16,17,18^

The most commonly reported complications in triple combined surgeries are graft failure and hypotony. Glaucoma and graft rejection occur less frequently.^3,7,8,14,19,20^ In our case series, hypotony occurred in 3 eyes, graft failure in 2 eyes, and graft rejection in 1 eye. One eye developed PVR and another exhibited macular atrophy.

One of the most important causes of such discrepancies in the literature has been associated with the inclusion of eyes with varying severity of primary pathology and separate groups such as traumatic and nontraumatic cases. Some authors have suggested that postoperative complications observed in trauma cases are more related to the severe damage resulting from the primary trauma rather than the stress caused by combined surgery.^21^ They cited their cases in which positive outcomes were attained despite undergoing more invasive procedures as support for this hypothesis.^21^ In another study supporting this hypothesis, OTC rupture or Zone 3 traumas, scleral lacerations larger than 10 mm, and ciliary body damage were determined to be poor prognostic factors.^22^

Conflicting results have also been reported regarding the prognosis of traumatic and nontraumatic cases. Gelender et al.^21^ found that trauma cases had a poorer prognosis in their small case series, whereas Garcia-Valenzuela et al.^8^ reported that prognosis was poorer in nontraumatic cases due to the more chronic disease course.

In the present case series, two of the traumas were penetrating and six were ruptures. For both types, the success rate was 50%. Trauma location was Zone 3 in five cases and Zone 2 in three cases. Anatomic reconstruction was achieved in only one (20%) of the Zone 3 cases (20%) and all of the Zone 2 cases. The relatively poor prognosis of Zone 3 cases in our series was consistent with the literature, but we observed no significant correlation between success rate and whether the trauma cases were rupture or penetration.

Another factor reported to impact surgical success is postoperative contact of the silicone oil tamponade with the corneal endothelium.^23,24^ Type of tamponade used in case series and differences between cases in terms of tamponade permanence and lens status may be other causes of the discrepant results found in the literature. In this study, silicone tamponade was used during surgery in all cases and was not removed from three eyes that were prephthisic. However, we did not encounter graft failure due to silicone oil/endothelium contact in our case series. In some of our patients, IOL prevented silicone/endothelium contact, while in other cases the silicone oil was removed before any damage occurred. In this respect, we believe that the small number of aphakic cases (37.5%) had a positive impact on prognosis.

Due to the risk of graft failure associated with long-term silicone exposure and aqueous humor deficiency, Chen et al.^25^ suggested suturing the removed corneal tissue back in place rather than placing an allograft in the same session, then performing PK in selected eyes with IOP higher than 8 mmHg. However, in their study, 62% of the eyes developed persistent hypotony and 13.5% had indications for enucleation. They reported that anatomic correction was achieved in only 15 eyes (20%).

Persistent corneal edema is one of the most important poor prognostic factors in combined surgeries. In their large series of 34 trauma cases, Roters et al.^23^ reported phthisis in 8 eyes, hypotony in 10 eyes, and graft failure due to silicone/endothelium contact in 21 eyes. In eyes that definitely did not have graft rejection, decreased aqueous humor secretion, inadequate feeding of the corneal endothelium due to low hydrostatic pressure, and postoperative inflammation have been implicated in the etiopathogenesis.^26,27,28^ In this regard, hypotony is a risk factor on its own. Three eyes in the present study had corneal edema, two of which also exhibited hypotony. A 16-year-old patient with corneoscleral rupture in Zone 3 did not have hypotony but early graft rejection was observed at postoperative 3 months.

In such cases, timing of the surgery is another controversial issue. Roters et al.^23^ recommended postponing the surgery to promote graft survival. They speculated that the risk of graft rejection is higher in surgeries performed within the first 8 months after trauma, and recommended that PK surgery be postponed until ocular inflammation subsides. However, inability to visualize the retina in postoperative follow-ups is an important drawback of this approach. Lee et al.^29^ reported that performing surgery within the first month improved prognosis in terms of retinal reconstruction. In our study, the mean interval between trauma and surgery was significantly shorter in successful cases.

Endoscopic PPV is performed as another alternative to temporary keratoprostheses. Studies comparing the two methods have shown that outcomes are similar in terms of anatomical and functional success. Endoscopic surgery was found to be superior in terms of the shorter time to diagnosis and surgery.^17,19^ Disadvantages of endoscopic surgery are that it does not allow for bimanual surgery, does not provide stereopsis, and is costly.^6^

### Study Limitations

Limitations of our study are its retrospective design and small number of cases. However, our results suggest that the combined triple procedure of PPV and PK with temporary keratoprosthesis enables intervention in a single surgical session as a last chance for anatomic and functional rehabilitation of eyes which would otherwise be considered inoperable. In such cases, patient selection is extremely important and patients must be well informed about the prognosis.

## Conclusion

In these cases, saving the eye seems to be a more realistic goal than improving vision. Although it depends on the severity of the preoperative findings, prognosis appears to be better than the natural disease course.

## Figures and Tables

**Table 1:**
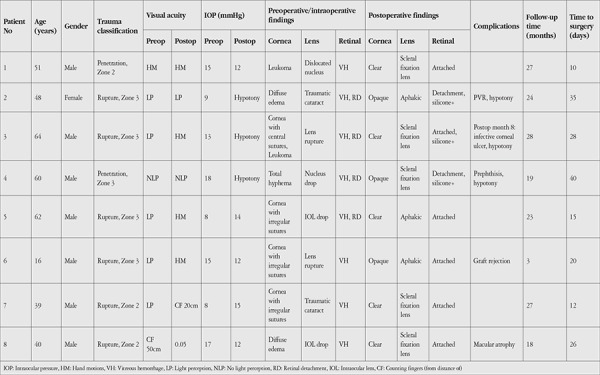
Detailed demographic and ocular features of the patients

**Table 2:**
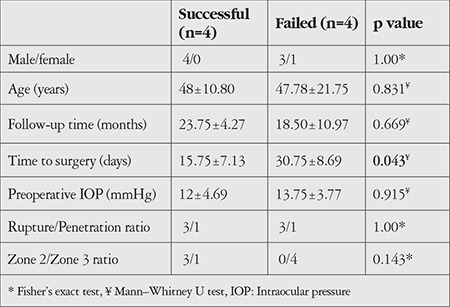
Demographic and ocular features of successful and unsuccessful cases
